# Human Chorionic Gonadotropin Priming Does Not Improve Pregnancy Outcomes of PCOS-IVM Cycles

**DOI:** 10.3389/fendo.2020.00279

**Published:** 2020-04-30

**Authors:** Yihua Lin, Xiaoying Zheng, Caihong Ma, Xiaoxue Li, Xinyu Zhang, Puyu Yang, Jiayu Xu, Jinliang Zhu

**Affiliations:** ^1^Department of Obstetrics and Gynecology, Center for Reproductive Medicine, Peking University Third Hospital, Beijing, China; ^2^National Clinical Research Center for Obstetrics and Gynecology, Beijing, China; ^3^Key Laboratory of Assisted Reproduction, Ministry of Education, Beijing, China; ^4^Beijing Key Laboratory of Reproductive Endocrinology and Assisted Reproductive Technology, Beijing, China

**Keywords:** HCG, IVM, PCOS, oocyte maturation, clinical pregnancy

## Abstract

**Background:** Influence of pre-retrieval human chorionic gonadotropin (HCG) priming on outcomes of *in vitro* maturation (IVM) remains controversial. This study aimed to evaluate the effect of HCG priming before oocyte retrieval on clinical outcomes of IVM cycles in patients with polycystic ovarian syndrome (PCOS).

**Methods:** This was a retrospective cohort study analyzing data from the first IVM cycles of unstimulated PCOS patients in a reproductive center of university affiliated hospital from January 2006 to December 2017. Patients received HCG injection before oocyte retrieval were assigned to HCG priming group and those without HCG administration were categorized as none HCG priming (Non-HCG) group. Main outcomes included oocyte maturation rate, number of embryos available, clinical pregnancy rate, and live birth rate. Candidate factors of clinical pregnancy rate was explored by univariate analysis and multivariate logistic regression analysis.

**Results:** There were 324 patients meeting the inclusion and exclusion criteria. Among them, 129 women received HCG priming and 195 other did not. Women in HCG group had significantly lower basal FSH level (5.17 ± 1.63 vs. 5.80 ± 2.38) than Non-HCG group. Both FSH levels were <10 IU/L and the absolute difference was 0.63 IU/L. Other basic characteristics were similar between groups with or without HCG priming. Oocyte maturation rate was trend to be higher in HCG group (52.68 vs. 48.56%) but no statistical significance was found (*P* = 0.097). No significant difference in clinical pregnancy rate was found between HCG and Non-HCG groups (31.37 vs. 35.67%). Miscarriage rates (31.25 vs. 34.43%) and live birth rates were also similar between groups. HCG priming was not correlated with clinical pregnancy rate in both univariate analysis (*P* = 0.468) and multivariate logistic regression analysis (*P* = 0.538; OR = 1.212; 95%CI: 0.657–2.237).

**Conclusion:** HCG priming before oocyte retrieval may not improve clinical outcomes of IVM in patients with PCOS.

## Introduction

*In vitro* maturation (IVM) technology has been clinically used for more than a quarter century since Cha et al. (1) reported the first live birth from oocytes matured *in vitro* in 1991. Then Trounson et al. (2) found that immature oocytes retrieved from patients with polycystic ovary syndrome (PCOS) had the potential to become mature *in vitro* and develop into competent embryos, with which successful live births were resulted. IVM had been estimated to lead to more than 5,000 live births all around the world until 2015 (3). Comparing with traditional ovary-stimulated *in vitro* fertilization (IVF), IVM has great advantages including lower cost, simpler treatment and decreased risk of complication such as ovarian hyperstimulation syndrome (OHSS). IVM is in increasing need as there are more and more patients asking for fertility preservation because of cancers and leukemia (4). However, IVM has not become a conventional treatment for infertility because of the reported unsatisfactory oocyte maturation rate and developmental competence (5, 6). Obstetric and neonatal outcomes after IVM are also of great concern (7). As a recent study reported (8), for IVM cycles of patients with high risk of OHSS, the maturation rate was 62.5% and clinical outcomes of IVM were worse than IVF cycles. Discovering possible factors affecting clinical outcomes of IVM cycles will help to improve IVM strategy.

Researchers are keeping trying to improve clinical outcome of IVM through different methods. Sánchez et al. (9) found that a prematuration culture in medium with CNP prior to routine culture of COCs might improve oocyte quality and subsequent developmental potential. Other than culture system, attempting to make an amendment to clinical regimen is another way to try. As a potential influence factor of IVM outcomes, the effect of human chorionic gonadotropin (HCG) priming before oocyte retrieval remains controversial. Some researchers reported that the administration of HCG, as a mimic of pre-ovulatory luteinizing hormone (LH) surge in spontaneous menstrual cycle, might trigger the resumption of meiosis and nuclear maturation of immature oocytes.

In 2000, Chian et al. (10) first explored the effect of HCG injection before oocyte retrieval on clinical outcome of IVM cycles of PCOS patients. They found that priming of 10,000 IU HCG significantly increased oocyte maturation rate after IVM. Fertilization rate and cleavage rate were also improved. Although it was a RCT, the study was limited by the small sample size (24 cycles in total) and low maturation rate (4.9%) of control group. So far published data about the correlation between HCG priming and IVM outcomes is scanty and controversial. Without clear recommendations, doctors of reproductive medicine can only choose to add or not to add HCG in IVM cycles according to their own experiences. Thus, this study aimed to evaluate the effect of HCG priming on clinical outcomes of PCOS-IVM cycles by analyzing the data collected in a single center from 2006 to 2017.

## Materials and Methods

### Patients

This is a retrospective cohort study approved by the Ethics Committee of Reproductive Medicine in Peking University Third Hospital. Data from IVM cycles performed in the Center for Reproductive Medicine of the hospital from January 2006 to December 2017 were reviewed. Patients were diagnosed as PCOS following the Rotterdam consensus criteria (11) and only their first IVM attempts without ovarian stimulation were included. The exclusion criteria were as following: female age >40 years old, PGD cycle, female or male abnormal chromosomal karyotype.

### IVM Protocol

IVM was conducted as previously described (12). In brief, a transvaginal ultrasound scan was conducted on day 2–3 after the onset of menstrual bleeding to exclude the existence of ovarian cysts. Growth of follicles were monitored by ultrasound on day 6–8 to exclude the development of a dominant one. Oocyte retrieval was scheduled once the endometrial thickness reached 6 mm and no follicle was larger than 10 mm in diameter. For priming patients, a dosage of 10,000 IU HCG was administrated subcutaneously and immature oocytes were collected 36–38 h later. For patients without HCG injection, oocytes were retrieved directly. Upon aspirated from small follicles, cumulus-oocyte complexes (COCs) were transferred into IVM medium (Sage IVM media kit, Origio, Denmark) to be cultured at 37°C in humidified air containing 5% CO_2_ for 28–32 h. All oocytes were then denuded from cumulus cells for maturity evaluation and mature oocytes were fertilized by intracytoplasmic sperm injection (ICSI) with sperms of husband. All zygotes were cultured in cleavage medium (G-M, Life Global, USA) supplemented with 10% synthetic serum substitute (SSS; Irvine Scientific, USA) in 5% CO_2_ incubator at 37°C up to day 3 after ICSI. D3 cleavage embryos were assessed according to the developmental stage and degree of cytoplasmic fragmentation before they were transferred or cryopreserved. No more than three cleavage embryos were selected for fresh transfer.

### Endometrial Preparation and Luteal Support

Patients were administrated with oestradiol valerate (Progynova, 6 mg orally, Schering, Berlin, Germany) from oocyte retrieval day for endometrial preparation. A dosage of 60 mg progesterone was injected from ICSI day. Medications were used daily until a negative pregnancy test or confirmation of clinical pregnancy. Serum hCG level was measured 13 days after embryo transfer. Clinical pregnancy was defined as the presence of an intrauterine gestational sac with fetal heart activity observed by ultrasound 30–35 days after embryo transfer.

### Data Collection and Statistical Analysis

Data about patients' basic characteristics, cycle characteristics (developments of oocyte and embryo) and clinical outcomes were collected. Patients were categorized as HCG group or Non-HCG group based on HCG primed before oocyte retrieval or not. Fertilization rate, cleavage rate, and available embryo rate were calculated per mature oocyte. Clinical pregnancy, ectopic, and live birth rate were calculated per embryo transfer cycle. Multiple pregnancy rate and miscarriage rats were calculated per clinical pregnancy. The comparisons of basic characteristics, cycle characteristics, and clinical outcomes were performed between groups. Then candidate variables for clinical pregnancy rate were estimated by univariate analysis and those were identified as being possibly significantly different between groups (*P* < 0.10) were further involved into multivariate logistic regression analysis model to explore potential confounding factors.

Statistical analysis was performed using Statistical Package for Social Science (SPSS) software, version 25.0 (IBM, Armonk, New York, USA). Student's *t*-test or Mann–Whitney *U*-test was used in the comparison of measurement data when appropriate. Comparisons between categorical data were performed using the Chi-square test. All reported *P*-values were two tailed, and *P* < 0.05 was established as the level of significance.

## Results

### Basic and Cycle Characteristics of Patients

Among 622 IVM cycles, 325 cycles were from the first IVM attempt without ovarian stimulation of PCOS women. One cycle was then excluded for female age >40 years. No PGD cycle or abnormal chromosomal karyotype of couples was found. A total of 324 cycles were included for analysis, in which the mean age of patients was (30.12 ± 3.67) years old. From all 5,361 COCs retrieved (16.55 ± 10.81 per cycle), 2,517 oocytes got matured *in vitro* (7.79 ± 5.32 per cycle) and the oocyte maturation rate was 46.95% (2,517/5,361).

There were 129 cycles in HCG group and 195 others in Non-HCG group. Basal FSH level was (5.17 ± 1.63) IU/L in HCG group and (5.80 ± 2.38) IU/L in Non-HCG group. The difference was statistically significant (*P* = 0.02). Age, BMI, and AFC were similar between the two groups (Table 1).

**Table 1:** Basic and cycle characteristics of patients in two groups.

**Variable**	**HCG group**	**Non-HCG group**	***P***
*N*	129	195	
Age, years	30.29 ± 3.65	30.02 ± 3.70	0.590^a^
Primary infertility, no. (%)	99 (76.7%)	144 (73.8%)	0.555^b^
Duration of infertility, years	4.83 ± 3.03	4.79 ± 2.80	0.742^a^
BMI, kg/m^2^	25.65 ± 4.09	24.98 ± 4.17	0.144^a^
Basal FSH, IU/L	5.17 ± 1.63	5.80 ± 2.38	0.020^a^
Antral follicle count	32.79 ± 12.32	30.97 ± 11.11	0.630^a^
No. of oocytes retrieved	15.74 ± 9.78	17.08 ± 11.46	0.320^a^
No. of mature oocytes	7.95 ± 5.61	7.69 ± 5.12	0.929^a^
Oocyte maturation rate (%)	52.68 ± 24.99	48.56 ± 22.25	0.097^a^
No. of fertilized oocytes	4.96 ± 3.70	4.74 ± 3.71	0.489^a^
Fertilization rate (%)	61.37 ± 25.34	62.30 ± 27.88	0.506^a^
No. of cleavage embryos	5.78 ± 4.27	5.78 ± 4.11	0.506^a^
Cleavage rate (%)	71.78 ± 24.17	75.06 ± 24.93	0.163^a^
No. of embryos available	3.04 ± 3.01	3.20 ± 2.96	0.305^a^
Available embryo rate (%)	37.70 ± 25.85	43.49 ± 27.57	0.090^a^

Numbers are mean ± SD unless otherwise indicated; No., number;

a Mann–Whitney U-test;

b *Chi-square test*.

### Clinical Outcomes of Patients in two Groups

Number of oocytes retrieved was similar between the two groups. Oocyte maturation rate was 52.68% in HCG group and 48.56% in Non-HCG group, reaching no statistical significance (*P* = 0.097). Fertilization rate and number of embryos available were also similar between groups. (Table 1).

**Figure 1:**
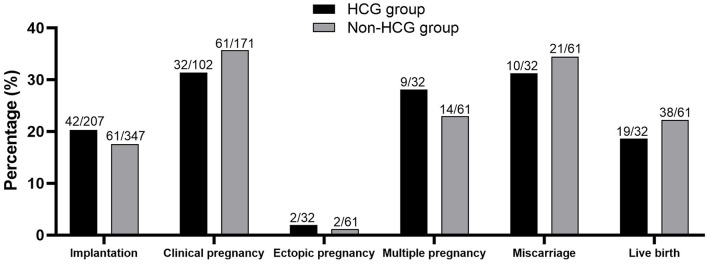
Clinical outcomes of patients in two groups. No significant difference was found between the two groups.

Embryo transfer was performed in 102 cycles of HCG group and 171 cycles of Non-HCG group. Endometrial thickness (7.72 ± 1.68 vs. 7.81 ± 1.52, *P* = 0.736) and number of embryos transferred (2.03 ± 0.52 vs. 2.03 ± 0.50, *P* = 0.995) were similar between groups. Clinical pregnancy rate, miscarriage rate, and live birth rate showed no significant difference between two groups. (Figure 1).

### Univariate Analysis of Factors on Clinical Pregnancy Rate

Of all 273 cycles with embryo transfer, 93 led to clinical pregnancies and the pregnancy rate was 34.07% in average. As for univariate analysis of factors on clinical pregnancy rate, among 18 variables tested by univariate analysis, 7 variables (number of fertilized oocytes, fertilization rate, number of cleavage embryos, cleavage rate, number of embryos available, available embryo rate, and number of embryos transferred) were, respectively, identified to be possibly significantly different between HCG and Non-HCG groups (*P* < 0.10, Table 2).

**Table 2:** Univariate analysis of factors on clinical pregnancy.

**Variable**	**Pregnancy**	**None Pregnancy**	***P***
HCG priming, % (no.)	34.4 (32/93)	38.9 (70/180)	0.468^b^
Age, years	30.32 ± 3.78	29.86 ± 3.57	0.509^a^
Primary infertility, % (no.)	76.3 (71)	74.4 (134)	0.731^b^
Duration of infertility, years	5.09 ± 3.01	4.81 ± 2.70	0.639^a^
BMI, kg/m^2^	25.08 ± 4.27	25.39 ± 4.16	0.532^a^
Basal FSH, IU/L	5.72 ± 1.51	5.40 ± 1.68	0.202^a^
Antral follicle count	33.68 ± 11.63	32.00 ± 11.47	0.285^a^
No. of oocytes retrieved	18.08 ± 10.78	17.18 ± 11.10	0.422^a^
No. of mature oocytes	8.49 ± 4.64	8.26 ± 5.42	0.367^a^
Maturation rate (%)	52.41 ± 21.37	51.75 ± 22.94	0.767^a^
No. of fertilized oocytes	5.95 ± 3.32	4.88 ± 3.66	0.002^a^
Fertilization rate (%)	72.49 ± 20.60	61.23 ± 24.04	0.000^a^
No. of cleavage embryos	7.00 ± 3.60	5.93 ± 4.11	0.007^a^
Cleavage rate (%)	83.11 ± 17.46	73.64 ± 20.88	0.001^a^
No. of embryos available	3.76 ± 2.66	3.34 ± 3.10	0.002^a^
Available embryo rate (%)	50.04 ± 26.37	43.19 ± 22.56	0.052^a^
No. of embryos transferred	2.16 ± 0.40	1.96 ± 0.54	0.002^a^
Endometrial thickness(mm)	8.00 ± 1.33	7.82 ± 1.67	0.495^a^

Numbers are mean ± SD unless otherwise indicated; No., number;

a Mann–Whitney U-test;

b *Chi-square test*.

### Multivariate Logistic Regression Analysis of Factors on Clinical Pregnancy Rate

These seven candidate variables, as well as HCG priming, were further involved into a multivariate logistic regression analysis model. The results showed that HCG priming before oocyte retrieval was not correlated with clinical pregnancy rate (*P* = 0.538; OR = 1.212; 95%CI: 0.657–2.237). Among all eight involved variables, larger number of embryos transferred was found to be associated with higher clinical pregnancy rate while other variables were not correlated with clinical pregnancy rate. (Table 3).

**Table 3:** Multivariate logistic regression analysis of factors on clinical pregnancy.

**Variable**	**β**	**Wald**	***P***	**OR (95%CI)**
HCG priming	0.193	0.380	0.538	1.212 [0.657, 2.237]
No. of fertilized oocytes	0.132	0.419	0.518	1.141 [0.765, 1.701]
Fertilization rate	0.408	0.063	0.802	1.503 [0.062, 36.455]
No. of cleavage embryos	0.054	0.084	0.771	1.056 [0.733, 1.520]
Cleavage rate	1.040	0.371	0.542	2.830 [0.100, 80.367]
No. of embryos available	−0.216	2.283	0.131	0.806 [0.610, 1.066]
Available embryo rate	1.418	1.108	0.293	4.129 [0.294, 57.917]
No. of embryos transferred	0.767	5.878	0.015	2.153 [1.158, 4.001]

*β, regression coefficient; OR, odd ratio; No., number*.

## Discussion

In traditional IVF treatment, the priming of HCG before oocyte retrieval has been routinely used and its role has been recognized. However, the effect of HCG priming in IVM cycles remains arguable. The present study found that cycle characteristics such as maturation rate and number of embryos available and clinical outcomes including clinical pregnancy rate, miscarriage rate, and live birth rate were all similar between HCG and Non-HCG groups. Although basal FSH level was significantly lower in HCG group (5.17 ± 1.63 vs. 5.80 ± 2.38, *P* = 0.02), both FSH levels were <10 IU/L and the absolute difference was only 0.63 IU/L. This might not influence the comparability of patients in two groups while other basic characteristics were similar.

In our study, although oocyte maturation rate seemed to be higher in HCG group (52.68%) than Non HCG group (48.56%), no statistical significance was found. Subsequent fertilization rate and number of embryos available were both similar between two groups. This result was different from previous studies. Chian et al. firstly (10) found that HCG priming before oocyte retrieval significantly improved oocyte maturation rate (48.2 vs. 4.9%). However, the extremely low oocyte maturation rate (4.9%) in control group implied that the results of the study two decades ago should be taken with caution. In another RCT held by Buckett et al. (13), although data about oocyte maturation was not shown, patients in HCG group were reported to have more embryos generated. We also conducted a RCT including 82 unstimulated PCOS-IVM cycles in 2012 (12). Within comparable basic characteristics, patients in HCG-primed group showed significantly higher oocyte maturation rate (55.43 vs. 42.29%). However, developmental competence of mature oocytes were similar between groups and no differences were found in embryo development.

There were explanations for the results. HCG was reported to influence oocyte maturation through LH receptor in granulosa cells. Only when granulosa cells become receptive to HCG stimulation can it stimulate steroid and extracellular matrix production (14). Then cumulus cells appear to be expansive. This is similar with the function of *in vivo* LH surge, which induces cumulus expansion of COCs. It has been found that dispersed cumulus cells (CCs) at the time of retrieval was associated with better maturation rate and embryo potential (15, 16). In our previous RCT study, about one-third of retrieved COCs in HCG group had dispersed CCs while all COCs in Non-HCG group had compact or sparse CCs. Thus, the maturation rate was higher after HCG priming.

However, unlike former studies, our present study found no improvement of maturation rate in HCG group. In fact, most of COCs retrieved in IVM cycles would be with compact CCs (12, 17). As the majority of follicles are in diameters <10 mm before recovery in IVM cycle, the granulosa cells are usually not responsive to LH, and neither to HCG. Thus, the priming of HCG before oocyte retrieval in IVM may not induce obvious effect on oocyte development. If larger follicles go over 10 mm, LH receptor expression may become stronger and the effect of HCG would become more predominant. This may also explain why embryo developments were not improved in our study and other researches (12, 13).

Clinical outcomes including clinical pregnancy rate and live birth rate per embryo transfer cycle were also similar between groups in the present study. Further, univariate analysis and multivariate logistic regression analysis of factors on clinical pregnancy rate also found no association between HCG priming and clinical pregnancy rate. The study of Chian et al. (10) was the only to report an improved clinical pregnancy rate (38.5 vs. 27.3%). Results of later researches were seldom consistent with it. Buckett et al. (13) reported that although patients in HCG group had more embryos transferred, implantation rate, and clinical pregnancy rate did not differ between groups with or without HCG priming. Our previous RCT (12) also found no increase in clinical pregnancy rate and live birth rate.

On one hand, although quantity of embryos transferred were compared, the quality of embryos was only evaluated morphologically, which might not reflect their true developmental potential. On the other hand, besides the quality of embryo, many other factors such as endometrial status were affecting the pregnancy and live birth. Buckett et al. (13) compared endometrial thickness, uterine artery pulsatility index and subendometrial blood flow between groups with or without HCG injection and concluded that HCG priming did not improve endometrial receptivity in IVM cycles. In other literature, comparability of only endometrial thickness did not represent the similarity of endometrial status. So, the effect of HCG priming on clinical outcomes needs to be interpreted with more evidence. A system review (18) included three RCTs reported no conclusive evidence that HCG priming had an effect on pregnancy, miscarriage or live birth rates in IVM. HCG priming might even reduce clinical pregnancy rate. However, the evidence quality was low because of the small number of data included.

Although the average clinical pregnancy rate (34.07%) and live birth rate (20.89%) of all included IVM cycles seemed to be lower than traditional fresh IVF-ICSI cycles (40.63 and 32.02%, respectively) during the same period in our center, they were worthwhile for patients who could not bear the risk of ovarian stimulation. Thus, IVM can play an important role in special cases, for instance fertility preservation of patients with cancer. Moreover, all embryos were transferred in day 3 cleavage stage in this study. If blastocysts were transferred, pregnancy rate and live birth rate might be more inspiring.

Miscarriage rate was 33.33% per pregnancy in the present study. It was considered as high comparing with 21.19% in traditional fresh IVF/ICSI cycles in the same period. Previous studies reported a miscarriage rate up to 57% in IVM cycles (12). Besides worse embryo potential, the influence of PCOS itself and dissatisfied endometrial receptivity were also usually suspected to be reasons. Women with PCOS was reported to have higher risks of pregnancy loss (19) and fetal chromosomal aberrations may be more frequent in PCOS patients than none PCOS women (20). Embryo transfer performed in fresh cycle may also be associated with pregnancy loss for the insufficiency of endometrial preparation. Walls and Hart (21) believed that freeze-all cycle could be a useful method to decrease miscarriage rate in IVM. The influences of PCOS and endometrial preparation on miscarriage rate of IVM cycle need to be determined by more well-designed studies.

To date published paper investigating the role of pre-retrieval HCG priming in IVM cycle were mostly conducted in unstimulated PCOS patients. FSH stimulation is widely considered to be a confounding factor. Dal Canto et al. (22) analyzed the priming of FSH and HCG on IVM cycles in a retrospective study. They found that oocyte maturation rate was significantly higher in expansive COCs (66.8%) than compact COCs (47.0%) and implantation rate of embryos from IVM oocytes (6.3–8.9%) was lower than oocytes matured *in vivo* (19.1%). Finally, patients with priming of both FSH and HCG had higher implantation rate and pregnancy rate than those with only HCG priming or without priming. This indicated that FSH priming may influence IVM outcomes. In the present study, we included only unstimulated PCOS patients to exclude the confounding effect of FSH. The results showed that HCG priming did not affect IVM outcomes without FSH stimulation. The effect of FSH on IVM outcomes will be explored in another study.

Studies exploring the effect of HCG priming on IVM outcomes is scanty. And three existing RCTs reported controversial conclusions. As Cochrane Database commented, quality of these evidence was low because of the small sample sizes. Our present study included 324 IVM cycles and analyzed the correlation between HCG priming and IVM outcomes in unstimulated PCOS patients. The findings may provide clinical practitioners with reference value to improve IVM protocol and give more evidence to the research field.

In conclusion, HCG priming before oocyte retrieval might not improve IVM outcomes in patients with PCOS. The results should be interpreted with caution for its nature of retrospective design and more large randomized control studies are needed to validate it.

## Data Availability Statement

The datasets generated for this study are available on request to the corresponding author.

## Ethics Statement

The studies involving human participants were reviewed and approved by Ethics Committee of Reproductive Medicine in Peking University Third Hospital. Written informed consent for participation was not required for this study in accordance with the national legislation and the institutional requirements.

## Author Contributions

JZ conceived and designed the study and revised the article. YL conducted acquisition, analysis, and interpretation of data and drafted the article. XZhe and CM conducted the interpretation of data and revised the article. XL, XZha, PY, and JX conducted the acquisition and analysis of data. All authors approved the final version of the manuscript.

## Conflict of Interest

The authors declare that the research was conducted in the absence of any commercial or financial relationships that could be construed as a potential conflict of interest.
